# Georgia Medical Association—An Abstract of the Proceedings

**Published:** 1871-05

**Authors:** 


*Georgia Medical Association—An Abstract of the Proceedings.*

The Association convened at Americus on the 12th of April.

The President not having arrived, Dr. J. G. Thomas, First Vice-President, took the Chair.

Dr. Hinkle, Chairman of the Committee of Arrangements, welcomed the Association in a beautiful address, tendering the cordial hospitalities of that pleasant little city, which were fully realized by members during their meeting.

To this the presiding officer, Dr. Thomas, responded in an appropriate address.

The Committee of Arrangements, which by the Constitution is made the Committee on Credentials, were delayed and somewhat confused in reporting the names of members present entitled to seats, by a protest from Fulton County Medical Society, against the reception of members of Atlanta Academy of Medicine, as members of the Association, which seems to have been placed in their hands. It was, however, finally overruled, and members of the Academy were admitted to seats.

When the organization was effected twenty-six new members were elected.

Members of the press were invited to seats, and the body adjourned till three P. M.

At the afternoon session, after prayer by Rev. Dr. Cooper, several resolutions were passed touching the eligibility of members, and other new members were elected, when the first business in order was called up, it being the consideration of the amended constitution laid over at last meeting in Macon, and made the first business in order for the session of 1871. It was read, and, after discussion, referred to a committee.

The protest of Fulton County Society against the Academy of Medicine was read, by Dr. Goldsmith, objecting to the admission of members of the Atlanta Academy of Medicine to seats in the Georgia Medical Association.

The prominent features of which are :

1. Because said Academy has members expelled from the Georgia Medical Association.

2. It has a member that was irregular in medicine by advertising, etc., and that Dr. Logan presented his name for membership.

3. The Academy elected a member who had contracted for a given sum, to treat the operatives of a manufacturing establishment.

4. The Academy had received as members, graduates of Atlanta Medical College, since the meeting of the Association in 1868 at Augusta.

5. Members of the Academy, and who are members of Georgia Medical Association, have violated that portion of the Code referring to "the duties of physicians to each other and the profession at large."

6. The Academy has allowed a journal, published by some of its members, also, to violate that part of the Code referring to the "duties of physicians to each other and to the profession at large."

Upon the charges and specifications given, the Fulton County Medical Society, by unanimous vote, arraign all the members of the Academy of Medicine, and appeals to the facts, regretting that its obligations require it.

(Signed) E. J. ROACH, *Secretary pro tem.*,
*Of Fulton County Medical Society.*

Dr. William O'Daniel was appointed Treasurer *pro tem.*, in the absence of the Treasnrer, Dr. Blackshear.

After a resolution to discuss the protest next morning, the Association adjourned to nine o'clock to-morrow morning.

---

APRIL 13, 9 O'CLOCK, A. M.

Dr. Campbell, the President, having arrived, took the Chair.

Other new members introduced.

Dr. Logan read a reply to the protest of yesterday, the prominent points in which are:

Does not recognize the right of the Medical Association to take cognizance of questions arising out of personal difficulties, nor the Fulton County Society as a regular body, not having the requisite number of members.

Only few votes in the Association excluding the members whose connection with the Academy of Medicine is objected to, and on a charge growing out of a purely personal difficulty, in compelling Dr. Powell to sever his connection with the College in Atlanta, and a difficulty with Dr. Crawford in the same institution.

These things did not impose the obligations of refusing to extend to Drs. Westmoreland, Miller, Johnson, O'Keefe and Armstrong the usual courtesies to other medical men.

Not appreciating the authority of said Doctors through the so-called Fulton County Society, he determined to continue the professional intercourse, though threatened with professional degradation by them.

The member objected to as having been irregular, had renounced such course and determined not again to engage in it, long before his connection with the Academy, which was ascertained by a committee before he was received. He was a useful surgeon during the war, and since his connection with the Academy has not violated any provision of the Code of ethics formed by the American Medical Association and adopted by the Academy of Medicine with which he is connected.

The member objected to on account of contracting with the manufactory has not been under such contract since his election, so far as has been ascertained.

As to fellowship with the graduates of Atlanta Medical College, no impropriety can exist, since the American Medical Association, at its meeting at Washington in 1870, virtually indorsed the standing of the College, and has never refused it representation. No objection to the old charter was made known to any organization, even by the said Powell, who had been connected under it for seven or eight years, until his summary separation from the College.

The Atlanta Medical and Surgical Journal, for which the Academy is held accountable, is in no manner controlled by it, nor connected further than to publish proceedings as any other print could do. Perfectly willing that these objectors should consider themselves leading medical men of Atlanta

In conclusion, it is clear that this Georgia Medical Association is not answering the purposes of its founders, or the high ends to which it should aspire. One reason for this is the perversion of its efforts for the advancement of science to subordinate and petty questions which should be left to local societies.

The introduction of the College question was injudicious, and unfortunate. The Association has been used by a few individuals in Atlanta for the purposes of revenge, etc.

The expulsion of some good men of Atlanta from this Association, emboldened the objectors to an attempt to regulate the profession and disturb the intercourse of gentlemen who had no connection with their quarrel.

Physicians in Atlanta and throughout the State will not allow this dictation by the self-constituted tribunal of Atlanta, made up of a small portion of the profession.

The action in Macon did not probably meet with favor from a great many there. Repeal that action and let the Association in future pursue the original designs, instead of allowing it used for selfish purposes.

Am in no way connected with the Atlanta Medical College, nor under any obligation to the Faculty (past or present), but shall "uphold the right though the heavens fall."

This reply was signed by J. P. LOGAN, M. D.,

*President of the Atlanta Academy of Medicine.*

Dr. Logan, after reading his reply to the protest of yesterday, offered the following:

WHEREAS, The controversy between the Faculty of the Atlanta Medical College, the Atlanta Academy of Medicine, the Fulton County Medical Society, or members of the same, who are members of this body, or between said bodies or individuals, and any other members of this Association are of a personal character, and ought never to have been introduced into this body, therefore,

*Resolved,* That all action of the Association upon these controvercies be *rescinded,* and be regarded as no longer a part of the archives of this body.

Dr. Nottingham offered the following:

*Resolved,* That all matters involved in the protest of the Fulton County Medical Society, and the reply of Dr. Logan thereto, be referred to a committee of five, consisting of the Committee on Credentials (Committee of Arrangements), and two other members to be designated by the Chair, and that the committee be requested to report at the earliest practicable moment.

After discussion, meeting adjourned to three P. M. to-day.

---

APRIL 13, THREE O'CLOCK P. M.

After admission of other new members, and referring to a committee the application of Dr. Walsh for membership, discussion was had on Dr. Nottingham's resolution, which was finally laid on the table by a vote of 33 to 21.

Dr. Logan's preamble and resolution were then passed by a vote of 35 to 14.

Dr. Thomas offered a protest against the action had which, by a resolution offered by Dr. Logan was allowed upon the minutes.

Association adjourned to nine o'clock to-morrow.

---

APRIL 14, NINE O'CLOCK, A. M.

Dr. Myers tendered his resignation as Secretary, which was accepted, with a resolution of thanks for the faithful performance of his duties.

Dr. S. H. Stout was appointed Secretary *pro tem.*

On motion of Dr. Cooper, it was resolved that the letter addressed by the Faculty and Trustees of Atlanta Medical College to the meeting of this Association in Macon in 1870, be placed on the minutes of this meeting, immediately preceding Dr. Logan's resolution.

Special committee on the amended new constitution report unfavorably, and it, with all proposed amendments, referred to same committee to report at next meeting in 1872.

The Committee recommend some slight amendments to the present Constitution.

Dr. Hinkle offered a resolution amendatory of the Constitution, requiring the President to appoint one from each Congressional District, to serve as a tribunal for the trial of ethical and constitutional questions, which goes over to the next meeting under the rule.

Dr. Holmes presented a memorial intended for the Legislature, from several clergymen, asking aid in the establishment of an inebriate asylum.

Dr. Kirkscey offered a resolution recommending this enterprise to the favorable consideration of the Legislature, without sanctioning all the positions taken.

Referred to Special Committee.

The President announced that his address, which from his absence was not made at the proper time, would be delivered at the close of the proceedings this evening.

Resolutions of thanks were offered by Dr. Griggs, and passed by acclamation, to editors, proprietors of the hall, physicians and citizens of Americus, and railroad companies, for kindness to the Association.

By Dr. Logan, and referred to Commiteee on Constitution, a resolution, that in future the Association shall be constituted of graduates of regular Medical Colleges in good standing residing in the State of Georgia.

Dr. Kirkscey offered a resolution, which passed, recommending that five dollars should be the lowest price charged by physicians in medical examinations for life insurance.

Nominating Committee requested to be ready for nominations this afternoon.

Report of Treasurer received.

Report of Committee on Publication received.

The action of Savannah Medical Society on the subject of general vaccination was read, and remarks made by Dr. W. H. Cumming, giving his plan, etc., for carrying out this desirable object.

The subject was referred to a special committee, consisting of Drs. Logan, Griggs and Green.

Dr. Stout offered a resolution, which was passed, indorsing the action of Savannah Medical Society, in reference to State Laws touching Insanity.

Adjourned to three P. M.

---

APRIL 14, THREE O'CLOCK P. M.

Association met. President Campbell in the Chair.

Committee appointed to consider the subject of Inebriates' Asylum, report favorably, which was adopted.

Auditing Committee on Treasurer's report find it correct.

Dr. Nottingham offered a resolution that three be appointed to memorialize the Legislature to amend laws in regard to insanity.

The Committee appointed to investigate the subject of general vaccination report, asking the appointment of six members to co-operate with Dr. Cumming in furtherance of his object. Adopted, and Drs. Logan, Nottingham, Stanford, Harris, Carter and Green appointed.

Communications read from Drs. Gaillard, of Louisville Journal, and Westmorelands, of Atlanta Journal, tendering their journals for publications, for which thanks were voted the editors.

Atlanta, Rome and Columbus were named for the next meeting of the Association. Columbus was selected.

The Nominating Committee retired to report nominations for officers of the Association.

The following is their report, which was unanimously adopted:

*President.*—George M. McDowell, M. D., of Barnesville.

*First Vice-President.*—E. J. Kirkscey, M. D., of Columbus.

*Second Vice-President.*—W. A. Green, M. D., of Americus.

*Corresponding Secretary.*—W. C. Musgrove, M. D., of Brooks county.

*Permanent Secretary.*—S. H. Stout, M. D., of Atlanta.

*Treasurer.*—W. O'Daniel, M. D., of Twiggs county.

*Orator.*—A. W. Griggs, M. D., of West Point.

A resolution, offered by Dr. Hinkle, for the appointment of one from each Congressional District to memorialize the Legislature, asking to extend physician's lien to all property, not included in the homestead, was passed unanimously.

Dr. W. A. Green read a paper on "Hypodermic Medication," in which the subject was ably discussed. A resolution was passed ordering its publication.

A resolution was passed, offered by Dr. Logan, tendering thanks to Dr. Campbell for faithful services as President.

Dr. Campbell tendered his acknowledgments, and proceeded to the delivery of his Annual Address.

The Association adjourned *sine die*, and, escorted by a band of music, proceeded to a hall where a magnificent banquet was prepared.

